# Postchemoembolisation syndrome – tumour necrosis or hepatocyte injury?

**DOI:** 10.1038/sj.bjc.6601329

**Published:** 2003-10-14

**Authors:** S J Wigmore, D N Redhead, B N J Thomson, E J Currie, R W Parks, K K Madhavan, O J Garden

**Affiliations:** 1 Department of Clinical and Surgical Sciences (Surgery), University of Edinburgh, Royal Infirmary, SI Little France Crescent, Edinburgh EH16 4SA, UK; 2 Department of Radiology, University of Edinburgh, Royal Infirmary, SI Little France Crescent, Edinburgh EH16 4SA, UK

**Keywords:** hepatic artery chemoembolisation, adriamycin, lipiodol, hepatocyte injury, hepatic neoplasms

## Abstract

Transarterial chemoembolisation of liver tumours is typically followed by elevated body temperature and liver transaminase enzymes. This has often been considered to indicate successful embolisation. The present study questions whether this syndrome reflects damage to tumour cells or to the normal hepatic tissue. The responses to 256 embolisations undertaken in 145 patients subdivided into those with hepatocyte-derived (primary hepatocellular carcinoma) and nonhepatocyte-derived tumours (secondary metastases) were analysed to assess the relative effects of tumour necrosis and damage to normal hepatocytes in each group. Cytolysis, measured by elevated alanine aminotransferase, was detected in 85% of patients, and there was no difference in the abnormalities in liver function tests measured between the two groups. Furthermore, cytolysis was associated with a higher rate of postprocedure symptoms and side effects, and elevated temperature was associated with a worse survival on univariate analysis. Multivariate analysis demonstrated that there was no benefit in terms of survival from having elevated temperature or cytolysis following embolisation. Cytolysis after chemoembolisation is probably due to damage to normal hepatocytes. Temperature changes may reflect tumour necrosis or necrosis of the healthy tissue. There is no evidence that either a postchemoembolisation fever or cytolysis is associated with an enhanced tumour response or improved long-term survival in patients with primary or secondary liver cancer.

The majority of primary and secondary liver cancers are not treatable by surgical resection due to their advanced stage at presentation (Famer *et al*, 1994). Transarterial chemoembolisation (TACE) of liver tumours is a technique that has been developed for the treatment of liver tumours. The goal of treatment may be to downstage a tumour such that resection or transplantation might be possible, or to provide palliation for unresectable or multifocal tumours (Higuchi *et al*, 1994; Lehnert and Herfarth, 1996; Harada *et al*, 1996). The technique allows delivery of chemotherapy and embolic material to either a part of the liver or to the whole liver, and can be used in conjunction with other embolic agents to limit the exposure of the body as a whole to chemotherapy, thereby reducing toxicity. Treatment can be repeated a number of times to attempt to achieve tumour response. Almost all protocols for TACE result in a characteristic syndrome following embolisation, which occurs between 40 and 85% of the patients (Hwang *et al*, 1987; Lin *et al*, 1988; Bismuth *et al*, 1992; Groupe d'Etude de la traitment du Carcinome Hepatocellulaire, 1995; Takenaka *et al*, 1995; Chung *et al*, 1996; Lu *et al*, 1999). The syndrome is characterised by a swinging fever, right upper quadrant pain, elevation of the hepatic transaminase enzymes, aspartate amino transferase, alanine amino transferase and gamma glutamyl transferase. The significance of this syndrome is uncertain, but has been considered by some to represent tumour necrosis and a successful response to chemoembolisation (Hwang *et al*, 1987; Bismuth *et al*, 1992; Groupe d'Etude de la traitment du Carcinome Hepatocellulaire, 1995; Castells *et al*, 1995; Takenaka *et al*, 1995).

This assertion has been brought into question by a recent study in which the livers of patients who had undergone TACE prior to surgical resection of primary hepatocellular carcinoma were examined (Paye *et al*, 1999). This study found that the presence of a chemoembolisation syndrome was not indicative of tumour necrosis and might indeed represent liver cell damage. One limiting factor for the above report was that all patients studied had primary hepatocellular carcinoma. Transaminase enzymes are produced by normal hepatocytes, but also by cells that have undergone malignant transformation. The elevation of serum concentrations of these enzymes following TACE could equally represent damage to normal hepatocytes, or hepatocytes that have undergone malignant transformation.

The present study addresses the question of whether the component of the chemoembolisation syndrome, known as cytolysis, which is manifested by elevation of hepatic transaminases, represents injury to normal hepatocytes or tumour necrosis, by examining patients who have undergone TACE for hepatocyte-derived (primary hepatocellular carcinoma) and nonhepatocyte-derived tumours (metastatic). In addition, the association with the clinical side effects of TACE and the prognostic significance of the postchemoembolisation syndrome is studied both in terms of tumour responses and survival.

## MATERIALS AND METHODS

### Patients

Between 1988 and 1998, 145 patients underwent TACE for primary and secondary liver tumours at the Royal Infirmary of Edinburgh. Data were collected prospectively on these patients by a dedicated data manager. Patients were then followed for a minimum of 3 years. Survival data on these patients are complete.

### Technique of TACE

Prior to the procedure, each patient received 1 g cefotaxime intravenously. Preliminary angiography (coeliac axis, superior mesenteric artery and selective studies of the hepatic arteries) was performed in order to visualise the hepatic arterial anatomy, portal vein patency and the presence of any arteriovenous shunts. Angiography was carried out using a 7F gauge superior mesenteric or sidewinder catheter (Cordis Europa NV). The artery (or arteries) supplying the tumour area was selectively catheterised. Where selective delivery of chemotherapeutic agents could not be achieved using a standard angiographic catheter, a Tracker 3 F gauge catheter system (William Cook, UK) was used. A standard dose of 40 mg of adriamycin (doxorubicin) was delivered in combination with the oily medium Lipiodol, the two agents being mixed together prior to injection. In general, a volume of 10 ml of lipiodol was used, but, with large hypervascular lesions, an additional 5–10 ml of lipiodol was used. Following delivery of the chemotherapeutic agents, a particulate embolising agent, Contour (Merck Pharmaceuticals, UK), or Spongstan (Johnson & Johnson Medical Ltd, Skipton, UK) was introduced into the tumour-feeding vessels, in order to slow or occlude the flow to the treated area. Where tumour extent and anatomy permitted, all sites were treated in one session. Where there was extensive bilobar disease, one lobe was treated at each session. Repeat chemoembolisations were repeated at 3 monthly intervals and follow-up computer tomography (CT) scans obtained at 6–8 weeks following each procedure. Repeat chemoembolisation was not usually carried out where there was progressive extrahepatic disease, portal vein invasion or general deterioration of the patient.

### Assessment of tumour response

Tumour size was measured by the product of the two largest perpendicular diameters. Decrease in tumour size was defined as a greater than 25% reduction in the sum of the products of the largest perpendicular diameters of all lesions on CT scanning, and static disease as less than 25% change in measurable lesions for at least 2 months. Increase in the size of lesions or the development of new hepatic metastases was considered as the progression of disease.

For the purposes of this study, we have used the same definition of the postchemoembolisation syndrome as that by Paye *et al* (1999), that is, a rise in temperature of 38.5°C or more or an increase in alanine amino transferase (ALT) of more than 100 U l^−1^ and more than double the preprocedural value. We have avoided the use of postprocedural pain or nausea to define the syndrome, since these factors are more subjective and less easy to define.

### Statistical analysis

Results are presented as medians and ranges unless otherwise stated. Comparisons between nonparametric data were performed using the Mann–Whitney *U*-test or Pearson *χ*^2^ test, with continuity correction as appropriate. Survival curves were plotted using the Kaplan–Meier plots and one-way comparisons of survival were performed using the log-rank test. A multivariate analysis was performed for survival, based on responses to the first episode of chemoembolisation. This analysis was performed using a Cox proportional hazards model using a linear regression function. The time function was survival and this was censored. Covariates entered into the model were age (continuous), alkaline phosphatase (>240 U ml^−1^), elevated ALT (>100 U ml^−1^), bilirubin (*μ*mol l^−1^), albumin falling below 35 g l^−1^, temperature (>38.5°C) and the CT response of the tumour, classified as increase or new lesions, static, decrease or too unwell to be assessed (all entered as categorical variables). All statistical analysis was performed using SPSS 11.0, (Chicago, IL, USA).

## RESULTS

### Patient characteristics

The study population comprised 101 men and 44 women of median age 63 (30–82) years. The primary pathology was hepatocellular carcinoma in 75 patients (52%). All the remaining patients had metastatic secondary carcinoma confined to the liver. The most common primary site was the colon and rectum (52 patients). Cirrhosis was present in 58 patients (40%). The majority of these patients were those with hepatocellular carcinoma (55 patients; 95% of those with cirrhosis). There were two patients with Child-Pugh grade B cirrhosis, the remainder were all classified as Child-Pugh grade A.

### Technical aspects of TACE

All patients had a patent portal vein, and thus were eligible for TACE. Selective cannulation of the hepatic arterial branch supplying the liver was achieved in 228 embolisations (89%); in the remaining patients, the catheter was positioned in the common hepatic artery distal to the ostium of the gastroduodenal artery. Doxorubicin (adriamycin) 40 mg mixed with lipiodol 10–20 ml was delivered in 238 procedures (93%), with 20 mg being delivered in the remainder. The reason for using a lower dose in certain individuals was documented toxicity to this drug during the previous TACE.

### Responses to chemoembolisation

Elevation of temperature above 37.0°C occurred following TACE in 89% patients, and 41% of the patients developed a fever diagnostic of the postchemoembolisation syndrome (>38.5°C) (Table 1

**Table 1:** Biochemical and temperature responses to transarterial chemoembolisation in 256 embolisations undertaken in 145 patients

	**Pre**	**Post**	**Change**	***P***
Albumin	39	34	−5	0.0001
Aspartate aminotransferase	30	227	+158	0.0001
Alkaline phosphatase	153	182	+15	0.0001
Bilirubin	11	21	+11	0.0001
Prothrombin ratio	1.0	1.1	0	0.0001
Temperature	36.8	38.5	+1.7	0.0001

Results are expressed as medians, and statistical comparison between groups was performed using the Mann–Whitney *U*-test.

). Elevation of transaminases occurred following TACE in 93% of patients. There were other disturbances in serum biochemistry, evident with a fall in albumin of 5 g l^−1^, which corrected to normal values in majority of the patients within 1 week. Bilirubin increased slightly and International Normalised Ratio (INR) also increased, suggesting that TACE may have compromised the hepatic synthetic capacity (Table 1).

### Responses to chemoembolisation in hepatocyte-derived and nonhepatocyte-derived tumours

The magnitude of the responses to chemoembolisation in patients, based on the nature of the primary tumour, is shown in Table 2

**Table 2:** Biochemical and temperature responses to transarterial chemoembolisation in patients with primary hepatocellular carcinoma 125 embolisations undertaken in 75 patients and secondary carcinoma 131 embolizations performed in 70 patients

	**Overall change**	**Hepatocellular carcinoma change**	**Secondary carcinoma change**	***P***
Albumin	−5 (−12–5)	−5 (−15–+5)	−5 (−13–2)	NS
Aspartate aminotransferase	153 (−8–2309)	+151 (33–1423)	+188 (8–1717)	NS
Alkaline phosphatase	15 (−123–800)	+7.5 (335–1176)		NS
Bilirubin	12 (0–116)	+13 (−5–+116)	+10 (−5–123)	NS
INR	0 (−0.1–0.5)	+0.1 (−0.2–+1.1)	0 (−0.1–4)	NS
Temperature	1.6 (0–3.2)	+1.8 (0–3.2)	+1.6 (0–3.4)	NS

Results are expressed as medians and ranges, and statistical comparison between groups was performed using the Mann–Whitney *U*-test, NS=not significant.

. Patients with hepatocyte-derived hepatocellular carcinomas had a higher bilirubin and prothrombin ratio compared to those who had nonhepatocyte-derived carcinomas (secondary tumours). There was, however, no significant difference in the magnitude of the changes in response to chemoembolisation depending on the tumour type.

### Does response to chemoembolisation predict symptoms?

Specific symptoms attributed to chemoembolisation were reported by 57 patients. These were most commonly nausea and vomiting or pain. There was no relationship between postchemoembolisation syndrome and the frequency of postprocedure complications; however, there was an increased frequency of complications in patients who had evidence of cytolysis (Pearson *χ*^2^ with continuity correction=4.218, d.f.=1, *P*=0.04).

### Does the occurrence of postchemoembolisation syndrome or the presence of cytolysis predict either tumour response or outcome following chemoembolisation?

**Figure 1:**
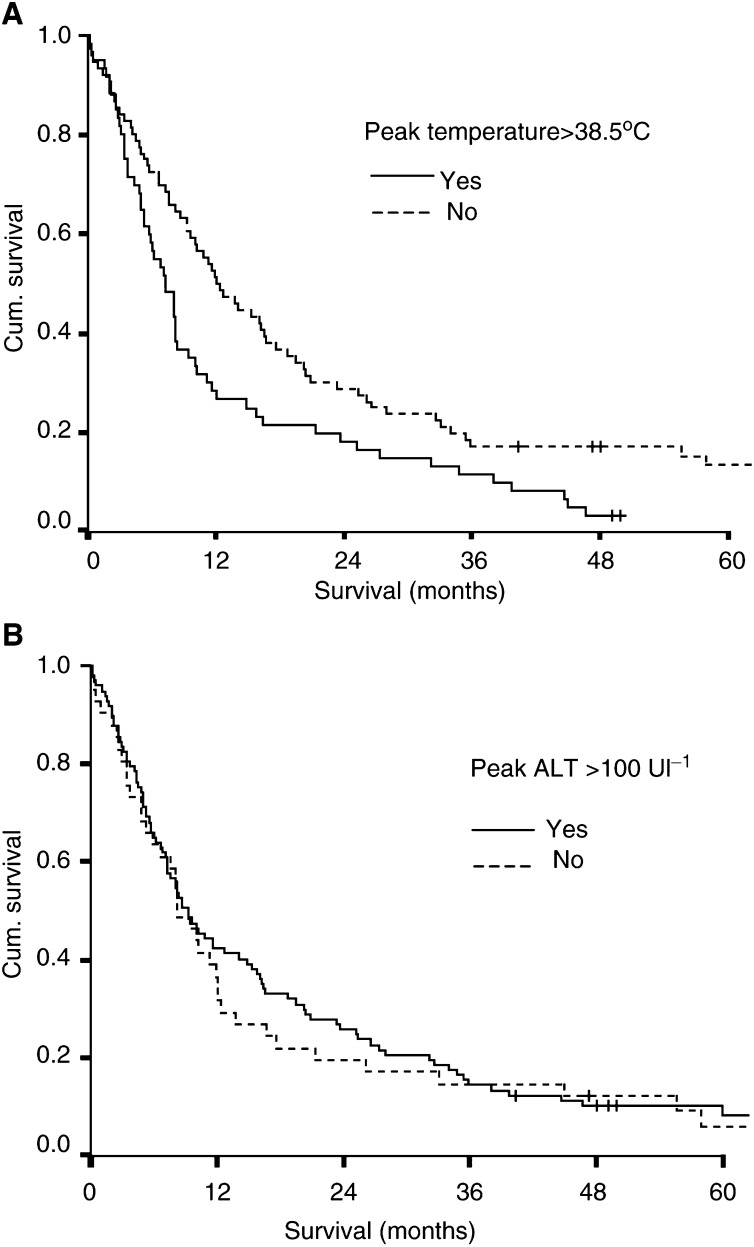
Survival curves of patients depending on (**A**) the presence or absence of postchemoembolisation syndrome (temperature >38.5°C, log rank=7.16, d.f.=1, *P*=0.0076) and (**B**) cytolysis (increase of serum AST of >100 U and more than 2 × prechemoembolisation value, log rank=0.2, d.f.=1, *P*=0.65).

Neither the postchemoembolisation syndrome (log rank=1.582, d.f.=3, *P*=0.663) nor cytolysis (log rank=2.86, d.f.=3, *P*=0.41) was associated with improved tumour response, based on CT assessment 8 weeks following chemoembolisation. Survival analysis demonstrated that the presence of postchemoembolisation syndrome was associated with an adverse outcome (log rank=7.16, d.f.=1, *P*=0.0076) (Figure 1a

). The presence of cytolysis had no effect on outcome (Figure 1b).

When subgroup analysis was performed, the presence of postchemoembolisation syndrome in those with primary liver tumours was associated with a poorer survival (median survival 6.8 months, range 4.7–9 months) compared to those without the syndrome (median survival 12 months, range 7.6–16.5 months) (log rank=4.44, d.f.=1, *P*<0.04). There was no significant difference in those with secondary tumours. Analysis of noncirrhotic patients with postchemoembolisation syndrome showed a poorer survival (median survival 8.2 months, range 7.9–8.5 months) compared to those without the syndrome (median survival 14 months, range 7.4–20.7 months) (log rank=4.6, d.f.=1, *P*<0.04). There was no significant difference in cirrhotic patients.

Using a Cox Proportional Hazards model, univariate and multivariate analysis of a number of factors was undertaken to assess their individual and combined impact on survival following the first episode of chemoembolisation. High bilirubin, alkaline phosphatase, temperature, low albumin, increasing age and a poor CT response to chemoembolisation were all associated with an adverse survival, on the basis of univariate analysis (Table 3

**Table 3:** Multivariate survival analysis using a Cox proportional hazards model, based on the response of patients to their first episode of chemoembolisation

**Co-variate**	**Univariate *P***	**Multivariate *P***
Age	0.01	0.029
High AlkPhos	0.0001	0.166
High bilirubin	0.0001	0.247
High ALT	0.654	0.425
Low albumin	0.0001	0.012
High temperature	0.008	0.766
Tumour response (CT)	0.0001	0.00001

). When multivariate analysis was performed, however, only age, low postprocedural albumin (<35 g l^−1^) and tumour response to CT scan remained significant. The response of the tumour based on CT scanning had the most significant effect and predicted outcome most accurately (Figure 2

**Figure 2:**
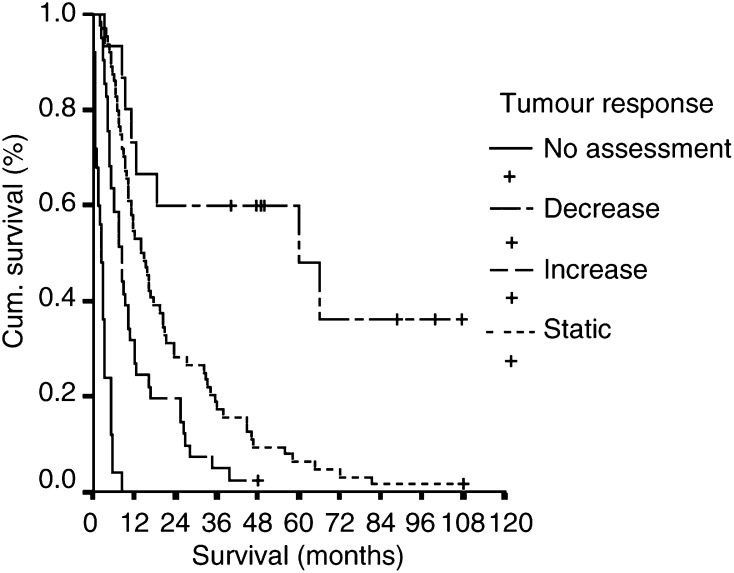
Survival of patients with liver cancer, based on their response to first treatment with TACE assessed by CT scan 6–8 weeks following the procedure (log rank=137.3, d.f.=3, *P*<0.0001).

).

## DISCUSSION

As a treatment modality, TACE retains a useful role in treating patients with primary or metastatic cancer, which is confined to the liver but which may not be suitable for resection or transplantation. It has been used as a primary therapy to try and downstage tumours prior to surgery and also in a palliative context. The prediction of who will benefit from this treatment and how to assess the response to it is rather more difficult. Between 40 and 85% of patients undergoing chemoembolisation experience a syndrome characterised by elevation of temperature and abdominal pain. These symptoms are typically associated with elevation of the hepatic transaminases. Recent evidence has suggested that some of the elements of this syndrome may be associated with damage to normal liver parenchyma rather than reflecting the conventional view that this was a representation of tumour damage and necrosis. For this reason, questions have been raised as to whether the postchemoembolisation syndrome represents a beneficial or an adverse response.

Paye *et al* (1999) examined the association between biochemical and temperature changes in patients undergoing preoperative chemoembolisation and pathological findings on subsequent resection specimens. They found that postchemoembolisation cytolysis with or without fever was more likely to develop in patients with minor fibrosis of the liver compared with those who had obvious cirrhosis. Furthermore, they found that the extent of tumour necrosis did not correlate with the development of postchemoembolisation syndrome. Their interpretation of these data was that cytolysis following chemoembolisation represented damage to normal hepatocytes. All of the patients in that study had hepatocellular carcinoma and necrosis of these cells might contribute to elevation of transaminases following chemoembolisation. The present study has therefore compared responses to chemoembolisation in patients with both hepatocyte-derived (primary hepatocellular carcinoma) and nonhepatocyte-derived tumours (secondary hepatic metastases). In the present study, it was found that the pattern and the magnitude of changes in biochemical variables and temperature were not significantly different, depending on the type of tumour. Thus, when chemoembolisation is undertaken for tumours that are not derived from hepatocytes, elevation in transaminases must represent damage to normal hepatocytes. This study supports earlier studies (Lin *et al*, 1988) by demonstrating that the magnitude of damage to normal liver cells is related to the procedure rather than the tumour type.

Next we investigated whether there was a correlation between symptoms described by patients and the presence of cytolysis or temperature changes. There was no association between temperature elevation and symptoms; however, patients who experienced cytolysis had a higher incidence of postprocedure symptoms. It is possible that this may in part reflect damage to the normal liver tissue.

Finally, the relationship between postchemoembolisation syndrome and cytolysis and survival was studied. High temperature was associated with an adverse outcome on univariate analysis, although this effect became nonsignificant when other factors were considered in a multivariate model. Cytolysis had no bearing on survival in either univariate or multivariate analyses.

The postchemoembolisation syndrome may be an inevitable consequence of embolisation of an artery, which may supply both tumour and normal liver tissue. Our policy has been to attempt the most selective cannulation of the hepatic artery possible, with the goal of reducing collateral damage to normal liver. Even after adopting such a policy and achieving selective hepatic artery cannulation in 89% of procedures, our patients still had a cytolysis rate of 93% and elevation of temperature >38.5°C in 41% of the patients. These rates are as high as other studies that have not adopted selective hepatic artery cannulation, and have delivered embolic material distal to the ostium of the gastroduodenal artery (Paye *et al*, 1998). There is a real danger of causing irreversible biliary damage through chemoembolisation and many series present other complications directly attributable to arterial occlusion.

It is our impression that the postchemoembolisation syndrome and cytolysis are certainly not associated with a good response of the tumour and do not predict improved survival. While there is no conclusive evidence that they are associated with a worse survival, it is possible that if cytolysis is a result of damage to normal hepatocytes this may be a detrimental effect, and could perhaps reduce the ability of patients to tolerate multiple chemoembolic procedures.
